# Substantial influence on solar energy harnessing ability by geometries of ordered Si nanowire array

**DOI:** 10.1186/1556-276X-9-495

**Published:** 2014-09-15

**Authors:** Zilong Wu, Ziyi Wang, Songyou Wang, Zhenyang Zhong

**Affiliations:** 1 State Key Laboratory of Surface Physics and Key Laboratory of Micro- and Nano-Photonic Structures (Ministry of Education) and Department of Physics, Fudan University, Shanghai 200433, China; 2 Key Laboratory of Micro and Nano Photonic Structures, Ministry of Education, Shanghai Engineering Research Center of Ultra-Precision Optical Manufacturing, Department of Optical Science and Engineering, Fudan University, Shanghai 200433, China

**Keywords:** Si nanowire array, Reflectance, Solar energy, TMM

## Abstract

The reflectance of the controlled periodic Si nanowire (NW) arrays is systematically explored, which characterizes the influence on the solar energy harnessing ability by the geometries of the NW. A unique dependence of the reflectance of the Si NW array on the diameter, the height, and the bending of the NW are disclosed. The solar energy loss caused by the reflection of the Si NW array exhibits the minimum for the NW with intermediate diameter and length. A plane-wave-based transfer-matrix method (TMM) simulation is performed, which is well consistent with the experimental results. Our results demonstrate the design principle to optimize the Si NW arrays for high-efficiency solar cells.

**PACS:**

81.07.-b; 78.67.-n; 81.16.-c

## Background

**S**emiconductor nanowires (NWs) have been of great interest for their innovative applications in optoelectronic devices, such as solar cells, lasers, photodetectors, and sensors [1-6]. In particular, due to the strong scattering of incident light within the NW array, solar energy loss due to the reflectance from the NW arrays is substantially reduced. This is crucial for the high-efficiency solar cells. Accordingly, the solar cell is one of the most promising applications of the semiconductor NWs, which has been proven to have the ability of harnessing more incident light with fewer materials than the traditional wafer-based solar cells [7]. This property satisfies the need for solar cells with higher efficiency at lower cost. Extensive investigations have been done for the application of semiconductor NWs on solar energy conversion. However, although the conversion efficiency of a single-NW solar cell has successfully exceeded the Shockley-Queisser limit [8], the efficiency of solar cells based on NW array is still not high enough for industrial application. One of the most significant reasons for the limited efficiency of the NW-array solar cells is associated with the lack of understanding on the exact influence of the NW geometries to the solar energy harnessing ability. The optimal geometries with regard to the period, the diameter, and the height of semiconductor NW array for solar energy harnessing have not been realized yet. Enormous efforts [9-23] have been devoted to study the influence of geometries on the solar energy harnessing ability of the semiconductor NW arrays. For quite small NWs, theoretical studies indicate that the diameter and the length of the NWs considerably affect the reflectance [9,10]. Monotonic decrease of the reflectance with the increase of the NW diameter was also observed [12]. The reflectance is also considerably affected by the slope of the NWs [16]. In addition, overall impacts of the diameter and the height on the properties of the NW-based solar cells were studied [7,11], which are associated not only with the light trapping in the NWs but also with the carrier generation and collection in the NWs. One advantage of the NW-based solar cells is that the NWs favor the decoupling between the light management and the carrier collection, which enables the independent optimization on the optical and the electrical properties of the NW-based solar cell. However, the systematic studies about the impact of the diameter and the height on the reflectance of the NWs have not been done. No comprehensive understanding on how to optimize the geometries of the NWs for the high-efficiency solar cells has been obtained.

In this paper, we report on the systematic studies of the reflectance of the controlled and ordered Si NW array. By the combination of nanosphere lithography and metal-assisted chemical etching (MACE), periodic Si NWs with desired period, diameter, and height can be readily obtained. The reflectance spectra of Si NW array with the different diameters and the different lengths are measured. The solar energy loss caused by the reflection is calculated based on the reflectance spectra to characterize the solar energy harnessing ability of the Si NW array. Unique dependence of the reflectance on the diameter and the height of the Si NWs are observed. A plane-wave-based transfer-matrix method (TMM) is applied to simulate the solar energy loss due to the reflectance of the Si NW array with different geometries, which agrees well with the experimental data. Our results demonstrate some significant guides to optimize the NW geometries for industrial application of ordered Si NW array in solar cells with the high efficiency.

## Methods

**Figure 1:**
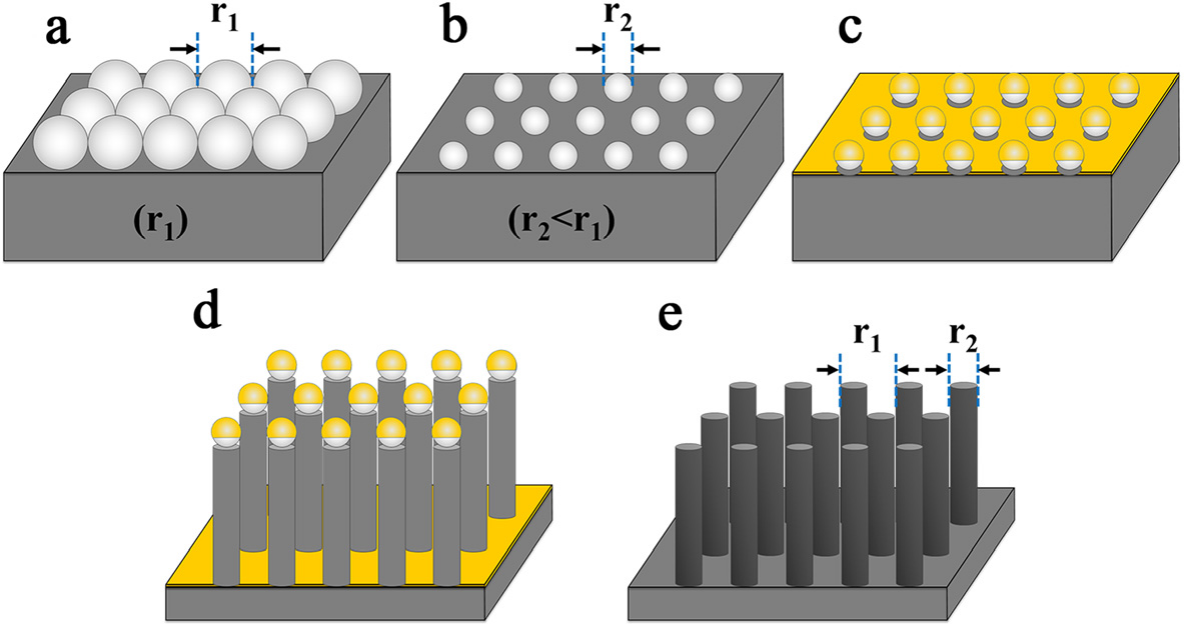
**Schematic illustration of the fabrication of ordered Si nanopillars. (a)** A close-packed monolayer of PS nanospheres on a clean Si substrate, **(b)** after RIE, **(c)** after Au deposition, **(d)** after the MACE, and **(e)** after the removal of the Au and the remained PS nanospheres.

The schematic illustration for the fabrication of ordered Si NWs is shown in Figure 1. Polystyrene (PS) nanospheres are first self-assembled on the Si (001) substrate, which are compactly arranged in a hexagonal lattice, as shown in Figure 1a. The PS nanospheres are then etched by a reactive ion etching (RIE) to shrink the diameter to desired size, as shown in Figure 1b. After that, an approximately 20-nm thick Au layer is deposited on the PS-covered substrate, as shown in Figure 1c. The Si underneath the Au is then etched by a mixture of 30% H_2_O_2_ and 40% HF (1:4 (v/v)). The solution keeps etching away the Si underneath the Au layer much faster than those under the PS nanospheres, which is so called MACE. As a result, Si NWs oriented along [001] direction are obtained, as shown in Figure 1d. The Au and Au-Si alloy are removed by a solution of I_2_:KI: H_2_O (1:4:40). To remove the remaining PS nanospheres, another RIE process is used. Finally, the ordered Si NW arrays are obtained, as shown in Figure 1e. The period of the Si NWs is exactly determined by the original diameter of the PS nanospheres. Accordingly, desired period of the Si NWs can be obtained by modifying the diameter of the original PS nanospheres. The diameter and the height of the Si nanopillars are subject to the time of the RIE and the MACE processes, respectively.

The geometries of the Si NWs are characterized using a scanning electron microscope (SEM) (Philips XL30 FEG, Royal Philips, Amsterdam, The Netherlands). The reflectance was measured by a PerkinElmer LAMBDA 950 UV/Vis/NIR spectrophotometer (PerkinElmer, Waltham, MA, USA) with an integrating sphere. The simulations of reflectance were conducted by a plane-wave-based TMM [24,25].

## Results and discussion

**Figure 2:**
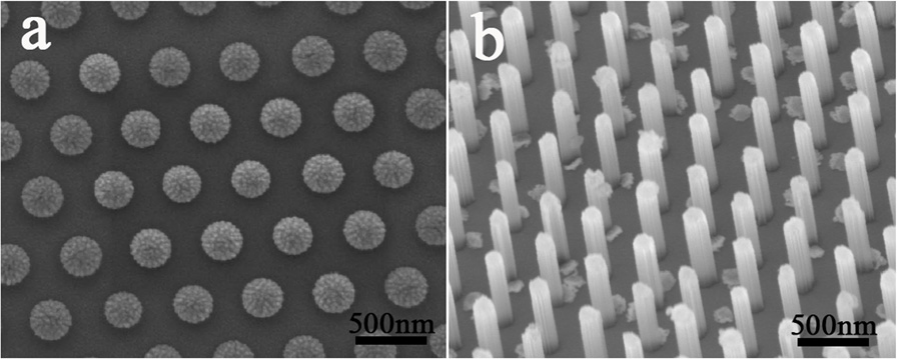
**SEM images. (a)** A typical PS nanosphere array. **(b)** The Si NW array after the MACE process.

Figure 2a shows the SEM image of ordered PS nanosphere array after the RIE process. The nanospheres are well arranged in a hexagonal lattice and quite uniform in size. Based on this patterned PS nanosphere array, the vertically aligned Si NWs with the (001) orientation are readily obtained after the MACE, as shown in Figure 2b. The ordering and the uniformity both remain well. The period and the diameter of the Si NW are controlled by the size of the original PS nanospheres and the condition of the RIE process, respectively. In addition, after the etching processes, the Si NWs appear like cylinders oriented along [001] direction without specific facets at sidewalls.

**Figure 3:**
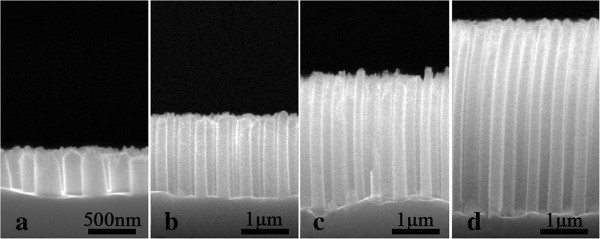
**SEM images of ordered Si NW arrays with different height. (a)** 0.54 μm, **(b)** 2.03 μm, **(c)** 3.35 μm, and **(d)** 4.59 μm.

The height of the Si NWs can also be readily controlled in the MACE process. Figure 3a, b, c, d shows SEM images of the Si NW arrays obtained with the etching times of 2.5, 8.5, 14.5, and 20.5 min, respectively. The corresponding heights of the Si NWs are about 0.54, 2.03, 3.35 and 4.59 μm, respectively. The period and the diameter of the Si NWs are 500 nm and 340 nm, respectively. It is found that the height of the Si NWs with the same period and diameter is almost linearly proportional to the etching time. Accordingly, the height of the Si NWs can be easily controlled by the etching time. The Si NWs remain well-ordered after a long etching time. Whereas, due to the effect of the liquid-surface tension force exerted on the NWs during the drying process of the sample [26], the Si NWs with a high aspect ratio (>10 in our case) tend to be slightly bended, as shown in Figure 3c, d. It is also worth noting that the bending angle increases with the height of the Si NWs.

**Figure 4:**
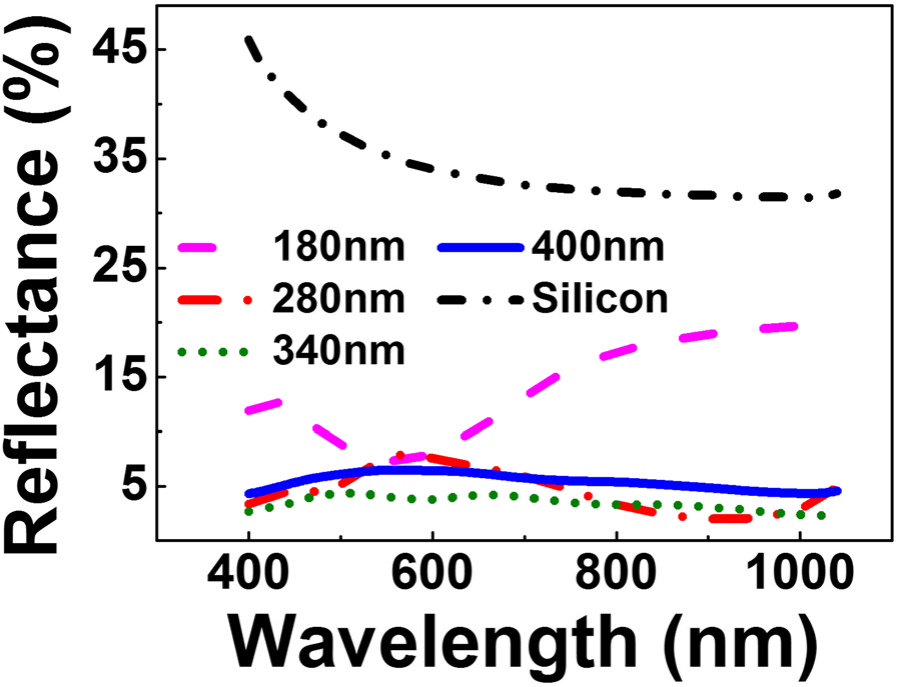
**Total reflectance of the Si NW array with different diameter.** The period and height of all the samples are 500 nm and 2 μm, respectively.

Figure 4 shows the spectra of the total reflectance of the Si NW arrays with diameters of 180 nm, 280 nm, 340, and 400 nm, respectively. The period and the height of the Si NWs in all the samples are 500 nm and approximately 2.03 μm, respectively. The total reflectance of a polished flat Si substrate is also shown in Figure 4 as a reference. It can be seen that the reflectance of the Si NW array is dramatically decreased in comparison with that of the flat Si substrate. In addition, the reflectance of the Si NW array shows a strong dependence on the diameter of the Si NWs. Moreover, such dependence is not monotonous. The Si NW array with the diameter of 340 nm exhibits the lowest reflectance, which remains lower than 5% throughout the wavelength range from 400 to 1, 000 nm. This result indicates that the Si NW array with the intermediate diameter tends to have the lower reflectance, which is consistent with the previous ones [7,14].

**Figure 5:**
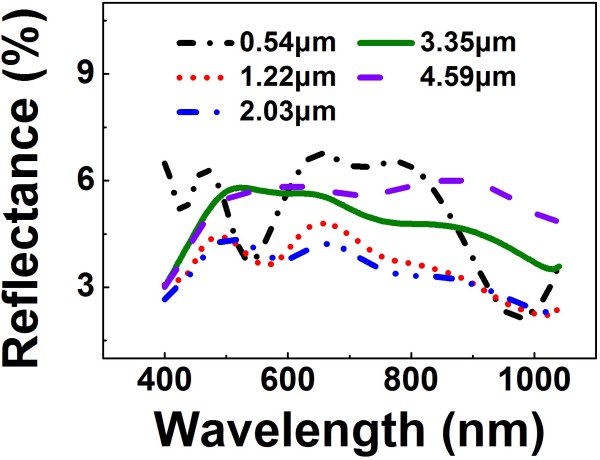
**Total reflectance of the Si NW array with different height.** The period and diameter of all the samples are 500 nm and 340 nm, respectively.

The total reflectance of the Si NW array is also considerably affected by the NW height. Figure 5 shows the total reflectance spectra of the Si NW arrays with the heights of 0.54, 1.22, 2.03, 3.35, and 4.59 μm, respectively. All the Si NW arrays have the same period (500 nm) and diameter (340 nm). Interestingly, the total reflectance tends to decrease first and then increase with the increase of the NW height. The lowest reflectance is observed from the Si NW array with the height of 2.03 μm. Such a dependence of the reflectance on the NW height is much different from the previous theoretical simulations [9,10,17].

**Figure 6:**
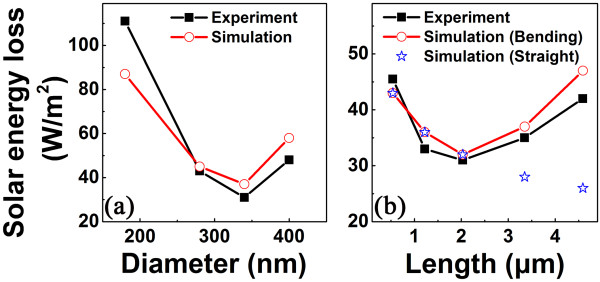
**SEL of Si NW array. (a)** versus the diameter of the Si NW, **(b)** versus the height of the Si NWs. The SEL for bending Si NW array is also plotted in **(b)**.

To further clarify the influence of the NW geometries on the solar energy harnessing ability of the Si NW array, the solar energy loss (SEL) caused by the reflection of the Si NW array is calculated according to the Equation 1,

(1) $$\text{SEL}=\overset{1000}{\underset{400}{\int }}I\left(\lambda \right)\alpha \left(\lambda \right)\mathrm{d\lambda}$$

where *I*(*λ*) is the solar energy intensity as a function of the wavelength *λ* at AM1.5 [27] and *α*(*λ*) is the measured reflectance of the Si NW array as a function of the wavelength. The SELs calculated from the measured reflectance spectra (seen in Figure 4) of the Si NW arrays with different diameters is shown in Figure 6a. Apparently, the SEL of the Si NW array tends to decease first and then increase with the increase of the NW diameter. The minimum SEL appears for the Si NWs with a diameter of approximately 340 nm. In addition, the SELs of the Si NW array with different heights of the Si NWs are also obtained, as shown in Figure 6b. It is clearly demonstrated that the SEL of the Si NW array tends to decrease first and then increase with the increase of the Si NW height. The minimum SEL appears for the Si NWs with the height of approximately 2 μm.

To understand these unique dependences of the reflectance on the geometries of the Si NWs, the TMM simulation is performed to simulate the total reflectance of the Si NW array, which has been proven to be very effective for dealing with the periodic NWs [9,24,25]. In the simulation, the incident light is normal to the Si NW array, and the scattered omnidirectional light is considered. Based on the simulated results, the SEL of the Si NW array with different diameter and height is then calculated, as shown in Figure 6a, b, respectively. The simulated SEL of the Si NW arrays with the different diameter is well consistent with the experimental one. Obviously, both the experimental and the simulated results show a similar dependence of the SEL of the Si NW array on the diameter. Interestingly, it can be found that the simulated SEL of the Si NW array monotonically decreases with the increase of the NW height, as denoted by open stars in Figure 6b, if all Si NWs are assumed to be straight. The simulated SEL of the Si NW array is remarkably smaller than the experimental one for the long and straight NW (e.g., longer than 2 μm); although for the short NW the simulated results agree well with the experimental ones, as shown in Figure 6b. A close inspection of the SEM in Figure 3c, d shows that the Si NW with the length longer than 2 μm is bent near the top. In addition, the bending of the NW becomes more pronounced for the longer one. Taking into account the bending of the long NW based on the SEM images, the simulated SEL then agrees well with the experimental one, as denoted by open circles in Figure 6b.

Such unique features of the solar energy harnessing ability of the ordered Si NW array are worth to be further exploration. The influence of the NW diameter on the anti-reflection ability of Si NWs has been studied experimentally by measuring specular reflectance [27]. The specular reflectance was found to increase with the diameter [28]. However, the total reflectance exhibits more complicated dependence on the diameter, which is consistent with the previous theoretical simulation based on the wave optics [10]. This result can be understood in terms of the effective medium approximation of the Si NW array. The Si NW array has been approximated to be an effective medium layer (EML) [13,16,19]. For the NWs with the small diameter, there will be an effective sharp interface between the EML and the substrate, where strong reflectance occurs. For the NWs with the large diameter, an effective sharp interface will appear between the air and the EML, leading to the strong reflectance. Accordingly, the reflectance should not be monotonically changed with the NW diameter. The minimum reflectance appears for the Si NW array with an intermediate diameter.

The dependence of the solar energy harnessing ability on the height of the Si NWs has also been reported by several groups. It was found that the SEL of the Si NW array decreases with the increase of the height of the Si NWs due to the increased light scattering in the nanostructures [9,14,17]. However, our experiments demonstrate the unique dependence of the SEL of the Si NW array on the NW height, which is considerably different from the previous one [9,14,17]. For straight NWs, the TMM simulations result in the decrease of the SEL of the Si NW array with increasing of the NW height, which are consistent with the previous experimental results [9,14,17]. On the other hand, the long Si NWs tend to be bent on the top in our cases. Taking into account the bending top of the long NWs, the TMM simulations of the SEL of the long Si NWs demonstrate an increase of the SEL of the Si NW array with increasing of the NW height, which agrees well with our experimental data. Such abnormal increase of the SEL from the long Si NW is mainly due to the bending geometry of the Si NW with a high aspect ratio (>10). The bending top of the Si NW can cause additional reflectance of the incident light. As the bending angle increases with the Si NW aspect ratio, the additional reflectance increases with the NW height, leading to the abnormal dependence of SEL on the NW height.

Our results clearly demonstrate that the solar energy harnessing ability of the Si NW array is intimately associated with the geometries of the NW. An insightful understanding on the effect of the NW geometries to the reflectance of the NW array is provided. According to our results, the optimized diameter and aspect ratio for the Si NW array with the periodicity of 500 nm is 340 nm and approximately 6, respectively. Such findings may help to design and fabricate novel solar cells with the high efficiency based on the optimized Si NW array.

## Conclusions

In summary, a feasible route to fabricate ordered Si NW arrays in a large area is developed. The systematic studies on the reflectance of the Si NW array are carried out. The unique dependences of the solar energy harnessing ability on the geometries of the Si NW are disclosed. These results are well explained based on the TMM simulation. Our results indicate that the geometries of the NW can considerably affect the performance of the NW-based solar cells. Our findings can serve as a guide to optimize the NW array for the fabrication of the high-efficiency solar cells.

## Competing interests

The authors declare that they have no competing interests.

## Authors’ contributions

ZLW prepared the samples and carried out the experiments. ZYW and SYW performed the TMM simulations. ZLW and ZYZ interpreted the results and wrote the manuscript. ZYW and SYW participated in manuscript preparation. All authors read and approved the final manuscript.
